# Sexually mediated phenotypic variation within and between sexes as a continuum structured by ecology: The mosaic nature of skeletal variation across body regions in Threespine stickleback (*Gasterosteus aculeatus* L.)

**DOI:** 10.1002/ece3.9367

**Published:** 2022-10-13

**Authors:** Heidi Schutz, Rebecca J. Anderson, Ethan G. Warwick, Tegan N. Barry, Heather A. Jamniczky

**Affiliations:** ^1^ Biology Department Pacific Lutheran University Tacoma Washington USA; ^2^ Department of Biological Sciences University of Calgary Calgary Alberta Canada; ^3^ Department of Cell Biology and Anatomy University of Calgary Calgary Alberta Canada; ^4^ Department of Biological Sciences University of Lethbridge Lethbridge Alberta Canada

**Keywords:** morphological evolution, morphometrics, mosaic phenotypic variation, sexual variation, skeleton, stickleback

## Abstract

Ecological character displacement between the sexes, and sexual selection, integrate into a convergent set of factors that produce sexual variation. Ecologically modulated, sexually mediated variation within and between sexes may be a major contributor to the amount of total variation that selection can act on in species. Threespine stickleback (*Gasterosteus aculeatus*) display rapid adaptive responses and sexual variation in many phenotypic traits. We examined phenotypic variation in the skull, pectoral and pelvic girdles of threespine stickleback from two freshwater and two coastal marine sites on the Sunshine Coast of British Columbia, Canada, using an approach that avoids a priori assumptions about bimodal patterns of variation. We quantified shape and size of the cranial, pectoral and pelvic regions of sticklebacks in marine and freshwater habitats using 3D geometric morphometrics and an index of sexually mediated variation. We show that the expression of phenotypic variation is structured in part by the effects of both habitat marine vs freshwater and the effects of individual sites within each habitat. Relative size exerts variable influence, and patterns of phenotypic variation associated with sex vary among body regions. This fine‐grained quantification of sexually mediated variation in the context of habitat difference and different anatomical structures indicates a complex relationship between genetically inferred sex and environmental factors, demonstrating that the interplay between shared genetic background and sexually mediated, ecologically based selective pressures structures the phenotypic expression of complex traits.

## Graphical abstract

This study was designed to examine the relative importance of sex in structuring phenotypic variation in stickleback fish. We used replicate examples of two habitat types (marine and freshwater) as a means of testing the hypothesis that the interplay between shared genetic background and sexually‐mediated selective pressures structures phenotypic expression of complex traits in a mosaic fashion. We found that sexual variation in the skull and girdles of sticklebacks does not manifest in a clear and uniform pattern of binary sexual dimorphism. Rather, it is a continuum of variation differing across structural units and reflecting the interacting effects of genetic sex and ecology on phenotypic variation.

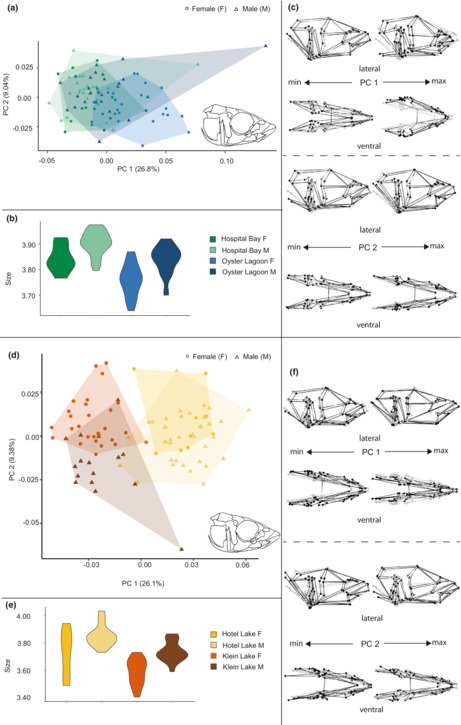

## 1 INTRODUCTION

Variation between genetic sexes occurs in many traits (e.g., body size, coloration and shape) and has long intrigued biologists. Studies of sexual differences in phenotype often focus on distinctly dimorphic traits within species (Berns, 2013), or traits such as male–male competition and female mating preferences (Darwin, 1859). As Lande (1980) noted, however, the integrated genetic influence on the phenotypic traits of both males and females produces correlated responses even in the face of strong selection on only one sex, or even divergent selection on both sexes (Fairbairn & Preziosi, 1994). Previous research shows that different male and female ecologies correlate with divergence between the sexes (Ronco et al., 2019; Temeles et al., 2010), and some studies point to this divergence as driven by both sexual selection and ecological differentiation (Butler & Losos, 2002; Slatkin, 1984). This phenomenon has been used to argue that ecological character displacement between the sexes and sexual selection formulate an integrated and convergent set of factors that produce sexual variation (De Lisle, 2019; De Lisle & Rowe, 2015). Indeed, ecologically modulated, sexually mediated variation both within and between sexes may be a major contributor to the amount of total variation that selection can act on in species (Aguirre et al., 2008; Bolnick & Doebeli, 2003; Butler et al., 2007). Sexual variation within and between sexes and adaptive speciation may go hand in hand, particularly with sexually mediated differential evolutionary pressures and occupation of different adaptive landscapes. For example, sex differences in red‐spotted newts have been linked with resource partitioning between the sexes (De Lisle & Rowe, 2015), and multiple selective pressures can simultaneously produce disruptive selection acting on complex traits, resulting in sexual variation driving population divergence (Cooper et al., 2011). Conversely, it has been argued that since sexual selection and ecological differentiation occur in an often integrated tandem, the shared genes controlling the phenotypic expression of the traits in question potentially mediate the drive to differentiate the sexes and serve as a limiting factor to both sexual variation and the overall variation of the species (Punzalan & Hosken, 2010).

Threespine stickleback fish (*Gasterosteus aculeatus*) are a powerful tool for exploring sexual variation. The species exhibits considerable number of sexually variable traits coupled with existence in a diverse range of aquatic habitats. These small (body length 5–11 cm) fish native to the coastal waters of the northern latitudes colonized saltwater, brackish and freshwater habitats in conjunction with Pleistocene glaciation events. Anadromous marine stickleback populations diverged to produce numerous isolated and phenotypically unique populations (Bell & Foster, 1994). This phenotypic diversity includes differences in armor, trophic morphology and body shape, among others (Hendry et al., 2013). Different stickleback morphotypes also display different levels of variation at the population level, with solitary lake forms displaying greater variation than marine or sympatric lake forms (Svanbäck & Schluter, 2012). However, coastal marine forms also exhibit genetic and morphological diversity (Leinonen et al., 2006). The capacity of *G. aculeatus* to rapidly diversify and adapt makes this species a model organism for studying evolutionary mechanisms and speciation (Bell & Foster, 1994; McKinnon & Rundle, 2002). In addition to their overall phenotypic diversity, sticklebacks exhibit considerable variation in the expression of sexually variable traits, including body shape and size, external plate number, head and fin bone length, and jaw shape and function (Reimchen et al., 2016). Sexual variation patterns also vary between wild and lab‐reared sticklebacks, and among and within ecosystems across a number of morphological features (Albert et al., 2008; Bell & Foster, 1994; Kitano et al., 2012; Leinonen et al., 2011; Spoljaric & Reimchen, 2008). Sexual variation and differentiation in whole‐body morphology of threespine sticklebacks is well documented (Albert et al., 2008; Kitano et al., 2007; Leinonen et al., 2011; Spoljaric & Reimchen, 2008). Although such studies provide considerable understanding of patterns of overall variation and difference in the entire stickleback body, understanding how individual (although highly developmentally and functionally integrated) morphological units vary and differ provides additional context for previously observed patterns.

In the present study, we focus on skeletal variation in the skull and girdles. Previous work demonstrates that overall skull shape and the shapes of individual skull elements vary widely both within and between habitats (e.g., (Barry, 2019; Jamniczky et al., 2015; Kimmel et al., 2012; Willacker et al., 2010). Sexually mediated shape variation in the skull is less well understood but is present in the whole skull (Pistore et al., 2016), with a particular focus on the trophic apparatus (Caldecutt & Adams, 1998; McGee & Wainwright, 2013). The threespine stickleback pectoral girdle contributes to locomotion (Webster et al., 2011) and functions in parental care (Künzler & Bakker, 2000). Prior work posits that variation in this structure correlates with habitat (Bell & Foster, 1994; Dalziel et al., 2012). Other studies document sexual variation in the size and position of the fin (Aguirre et al., 2008; Kitano et al., 2007). Additionally, the pectoral girdle's morphological plasticity appears to be seasonal and responsive to rearing environments (Hoffmann & Borg, 2006; Sharpe et al., 2008). The pelvic girdle, considered particularly well developed in sticklebacks (Bell & Foster, 1994), also exhibits considerable variability. Variation in its size and presence was hypothesized to be potentially associated with calcium availability (reduction/loss only occurs in freshwater environments (Klepaker et al., 2013) and predator presence/absence such that pelvic girdle dimensions become reduced in low calcium concentration environments and in the absence of predatory fishes (Bell et al., 1993). However, the involvement of the *Pitx1* gene is also well documented in most instances of pelvic girdle and fin reduction or loss in this species and the correlation between their absence and reduction with predators and calcium availability is not universal in the populations that exhibit this trait (Klepaker et al., 2013). Additionally, there is marked variation in pelvic fin morphology between marine and freshwater populations, where marine forms exhibit longer pelvic spines relative to freshwater forms (Bell et al., 1993). Pervasive sexual variability of the pelvic girdle also occurs, with females tending to have significantly longer pelvic bases than males and, in some populations, longer pelvic spines (Aguirre et al., 2008).

Many previous studies, although documenting important components of phenotypic variation, did not explicitly approach these phenotypes as consisting of complex traits that vary in all three spatial dimensions. Although 2D morphometric analyses based on photographs sample most of the form of fishes and are much more accessible and cost‐effective for sampling large numbers of individuals, the fine‐grained depictions of shape available from 3D analyses contribute essential information on patterns of variation that vary in three dimensions, such as those present in skulls and girdles. Further, much of the previous work aimed at elucidating differences among replicate habitats focused on the freshwater context, leaving variation among stickleback occupying marine habitats relatively poorly understood.

In this study, we examine overall morphological variation and sexually mediated variation in the skull, pectoral and pelvic girdles of threespine stickleback from two freshwater and two coastal marine sites on the Sunshine Coast of British Columbia, Canada, which belong to one of five Pacific genetic clusters of threespine stickleback and extend from Washington to Alaska (Morris et al., 2018) using a 3D approach. We note that, although the term ‘sexual dimorphism’ is pervasive in the literature and is often the focus of studies documenting sexual variation in phenotypes, considerable work demonstrates the abundance of overlap between the phenotypes assigned to genetically ‘female’ and genetically ‘male’ individuals (see MacLeod and Kolska Horwitz (2020) for a recent example). In this contribution, we explicitly avoid making any a priori assumptions about the presence of bimodal variation in phenotype. We ground our study in the premise that the expression of sexually mediated variation within and between sexes manifests in a range of ways, to assist in reducing bias and permitting exploration of how the total complement of phenotypic variation in a population under selection will structure adaptive change in that population and produce unique patterns. We predict that ecology and organism–environment interactions alter the expression of sexually mediated variation in complex traits such that different patterns of variation may be found in different habitats as well as among sites within replicate habitat types. Further, we predict that sexually mediated variation in multiple complex traits is present as a continuum along which genetically ‘male’ and ‘female’ individuals show both considerable within‐group variation and extensive overlap in the expression of these traits, rather than a bimodal distribution with well‐circumscribed phenotypes assignable to genetic sex.

## 2 METHODS

### 2.1 Specimen collection

Specimens were collected in the spring of 2015 and 2016 from the Sunshine Coast region of British Columbia, Canada, within the traditional territories of the Squamish, Sechelt, and Tla'amin and Klahoose nations. Two marine habitats along the Agamemnon Channel were sampled: Bargain Bay Lagoon (49°36′48.6″N, 124°1′46.9″W, *n* = 49) and Hospital Bay Lagoon (49°37′53.4″N, 124°1′48.0″W, *n* = 45). The approximate salinity in this region ranges from 20 to 32 ppt (Barry, 2019), and both habitats are tidally influenced. Two freshwater habitats in nearby Madeira Park were also sampled: Hotel Lake (49°38′18.8952″N, 124°2′48.9732″W, *n* = 47) and Klein Lake (49°43′50.1024″N, 123°58′27.732″ W, *n* = 44). Located approximately 1000 m from Hospital Bay Lagoon, Hotel Lake represents a shallow (~6 m) lacustrine environment in a populated setting. In contrast, Klein Lake, located approximately 12 km from Hospital Bay Lagoon, is approximately 36 m deep and considerably more remote.

Adult fish (determined by standard length ≥35 mm (Baker et al., 2015) were collected using minnow traps. Specimens were euthanized in the field using an overdose of Eugenol (Sigma‐Aldrich). Fin clips were collected and stored in 70% ethanol for genetic sex determination, and specimens were fixed flat in 10% neutral buffered formalin for 24 h and then moved to 70% ethanol for long term storage. Sampling was conducted in accordance with the standards of the Canadian Council of Animal Care, and all activities were approved by the Life and Environmental Sciences Animal Care Committee at the University of Calgary (AUPs BI09R‐41, AC13‐0040, AC12‐0057, AC16‐0059).

### 2.2 Genetic sex determination

Specimens were sexed by genotyping the isocitrate dehydrogenase locus following Peichel et al. (2004). Fin clip DNA was extracted using a DNEasy Blood and Tissue Kit (Qiagen) and stored at −20°C before use, and PCR products were visualized on a 2% agarose gel. Sex was assigned based on banding patterns as described by Peichel et al. (2004).

### 2.3 Computed tomography, modeling and landmarking

**FIGURE 1:**
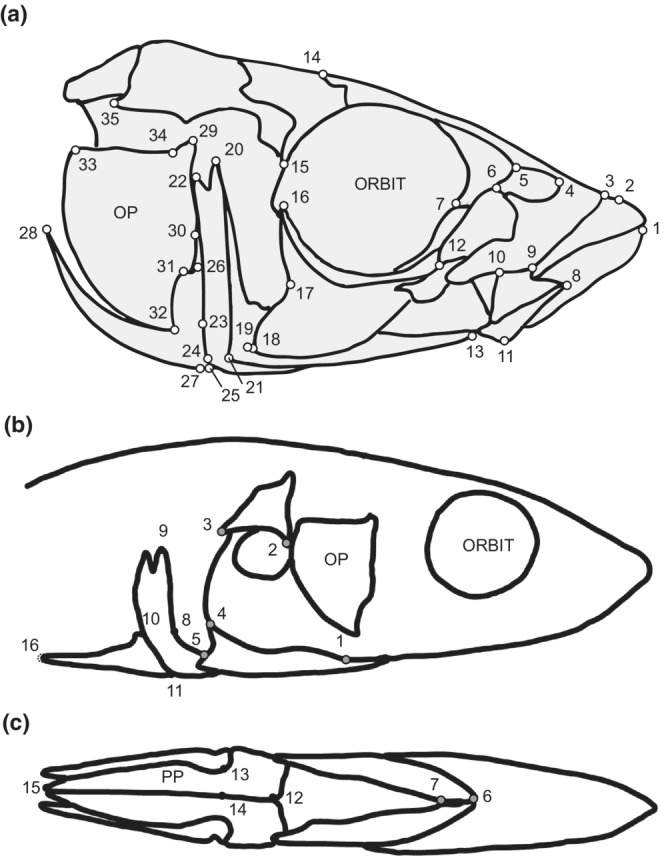
3D landmarks collected on the skull (white circles), pectoral (gray circles) and pelvic (black circles) girdles and shown in two views to best showcase landmark position. (a) Lateral view; (b) Ventral view. Landmarks on the pelvic spines (X) were removed following preliminary analysis. OP: Opercle. Landmarks adapted in part from Albert et al. (2008), Barry (2019), Bell and Foster (1994) and Morris et al. (2018). Skull landmarks: 1, anterior tip of dentary, 2, anterior tip of premaxilla, 3, anterior tip of maxilla, 4, anterior corner of nasal ventrolateral process, 5, dorsal corner of nasal‐lateral ethmoid suture, 6, dorsal maximum of lacrimal, 7, lacrimal‐prefontal suture on orbital, 8, anterior tip of articular, 9, ventral maximum of lacrimal, 10, dorsal tip of articular, 11, ventral‐most tip of articular, 12, lacrimal‐second orbital suture, 13, anterior tip of preopercle, 14, dorsal‐most extent of supraorbital, 15, ventral‐most tip of sphenotic, 16, dorsal‐most tip of third suborbital, 17, posterior minimum of third suborbital, 18, ventral‐most tip of third suborbital, 19, anterior minimum of preopercle, 20, anterior dorsal‐most tip of preopercle, 21, posterior maximum of preopercle, first ridge, 22, posterior dorsal‐most tip of preopercle, 23, dorsal‐most tip of interopercle, 24, ventral maximum of preopercle, second ridge, 25, ventral‐most tip of interoperculum, 26, dorsal‐most tip of subopercle, 27, ventral maximum of subopercle, 28, posterior tip of subopercle, 29, dorsal‐most tip of opercle, 30, anterior maximum of opercle, 31, anterior minimum of opercle, 32, ventral‐most tip of opercle, 33, posterodorsal tip of opercle, 34, opercular hinge angle, 35, posterior tip of pterotic. Pectoral and pelvic girdle landmarks: 1, anterior junction between ectocoracoid and coracoid at the caudal‐most projection of the coracoid foramen, 2, cranial‐ and dorsal‐most maximum of curvature of cleithrum on the inferior edge, 3, caudal‐most extension of the cleithrum, 4, dorsal caudal‐most extension of ectocoracoid, 5, posterior extension of ectocoracoid, 6, anterior tip of ectocoracoid, 7, posterior‐most point of the anterior contact between left and right ectocoracoid 8, anterior caudal‐most curvature on anterior process of pelvic plate at junction with ventral base of ascending branch of the pelvic plate, 9, dorsal most tip of ascending branch of pelvic plate, 10, dorsal most intersection between pelvic spine and ascending branch of the pelvic plate, 11, ventral most intersection between pelvic spine and ascending branch of the pelvic plate, 12, medial edge of cranial most point of the anterior process of the pelvic plate, 13, intersection between ventral point of pelvic spine and anterior process of the pelvic plate, 14, medial most point of junction between the anterior process and posterior processes of the pelvic plate at trochlear joint, 15, posterior tip of posterior process of the pelvic plate, 16, caudal tip of pelvic spine.

Specimens were subjected to micro‐computed tomography using either a Scanco uCT35 (Scanco Medical AG) or a Skyscan 1173 High Energy MicroCT (Bruker) instrument. Fish were scanned from the snout to the distal tips of the pelvic spines at a resolution of 20 μm using standardized parameters (70 KvP, 114 μA). Fish were wrapped in plastic in a standardized position with spines and fins flat against the body wall and mouths closed, and packed in foam to prevent any movement during scanning. Raw data were reconstructed into image stacks and output from each system in either .aim (Scanco) or .tif (Skyscan) formats and then imported into Amira v. 5.4 (ThermoFisher Scientific EM Solutions), where the Isosurface tool was used to produce a three‐dimensional mesh representation of the skeleton. Landmarks with XYZ coordinates were collected from 178 specimens: 70 from the skulls (Figure 1a), 14 from the pectoral girdles (Figure 1b), and 18 from the pelvic girdles (Figure 1c). Landmarks were collected in Amira and exported for further analyses in R.

### 2.4 Phenotypic analysis

All analyses described below were conducted using R v. 4.1.3 (RStudio Team, 2020, 2020), running in RStudio v. 2022.02.1 (RStudio Team, 2020). Analyses and plots were produced using base R as well as the following packages: geomorph v. 4.0.3 and RRPP v. 1.2.3 (Collyer & Adams, 2018), ggplot2 v. 3.3.5 (Wickham, 2016), plyr v. 1.8.6 (Wickham, 2011), and vegan v. 2.5‐7 (Oksanen et al., 2019). The annotated code is available as a file uploaded to Dryad.

### 2.5 Composition of the final dataset

Each landmark set (pectoral, pelvic, skull) was independently aligned using Generalized Procrustes Transformation (GPA; Dryden & Mardia, 1998) to remove the effects of scale, rotation and translation. These data were then examined for the presence of outlier landmarks, as determined by extreme positional variability. This procedure led to the removal of two landmarks located on the pelvic spines (Figure 1b; open circles), leaving 16 on the pelvic girdle. The remaining data were then realigned using GPA, and each dataset was assessed for the presence of outliers using the plotOutliers function in geomorph. This function identifies specimens that fall above the upper quartile in their Procrustes distance from the mean shape. No outlier specimens were found in any of the datasets based on this method, and we therefore chose a conservative approach and removed none. The composition of the final dataset is presented in Table 1. These aligned datasets were used for further analyses.

**TABLE 1:** Final dataset for phenotypic analysis

	Fresh	Marine	Hotel Lake	Klein Lake	Bargain Bay Lagoon	Hospital Bay Lagoon
Female	37	37	9	28	14	23
Male	53	52	37	16	26	26
Total	90	89				

*Note*: Numbers represent sample sizes.

### 2.6 Principal components of variance

Each landmark set was subjected to principal components analysis (PCA) in geomorph, to identify linear combinations of landmark coordinates responsible for the major axes of variation among individuals present in the complete dataset. Additional PCAs were performed for each habitat separately, with components and principal component (PC) scores retained from these analyses based on the broken stick model (Jackson, 1993; Legendre & Legendre, 2012), and implemented using the bstick function in the vegan package and used for statistical modeling of shape and size. PCs were also used to conduct further statistical analyses (see below) and to aid in visualization of the range of variation present at each site within a habitat.

### 2.7 Statistical models

Linear models were constructed for each dataset and sequentially evaluated for statistical significance, using high‐dimensional Procrustes Analysis of Variance with randomization of residuals using permutation (RRPP; Collyer et al., 2015) to investigate variation in size and shape between habitats and sexes, and the possibility of interactions between size and shape. Shape was included in these models represented by PC scores (see above), and size was included as a covariate and represented as the natural log of the centroid of the landmark configuration for each dataset. Models of increasing complexity were sequentially compared to determine best fit using multivariate ANOVA in the lm.rrpp package, where the null hypothesis of common allometry among habitats and sexes was rejected if a significant interaction between size and habitat was detected. If a significant main effect of habitat was detected, each habitat was then assessed separately following a new superimposition and PCA as described above to determine if sexual variation was site‐dependent within habitat. All models used Type III sums of squares, and the statistical significance of all comparisons in this section was determined via the nonparametric procedure in RRPP, using 10,000 permutations. 3D datasets provide additional information on complex shapes, but due to the complexity of their acquisition both with regard to cost and time as well as their production of a high number of variables, statistical methods that can accommodate datasets where variables outnumber observations are needed. Permutation procedures such as those available in RRPP are an effective means of managing such datasets where acquisition of larger sample sizes and the reduction of variables is simply not pragmatically possible (Collyer & Adams, 2018). We note that modeling site within habitat as a nested random effect may provide an alternative approach to this analysis; however, our sample size does not provide sufficient statistical power for this approach and we have thus chosen the simpler analysis described above.

### 2.8 Index of sexual dimorphism

The index of sexual shape dimorphism developed by Schutz et al. (2009) was used to quantify the relative amount of differentiation between genetically identified males and females from each habitat in this dataset. Briefly, the index compares the squared Procrustes distances for two groups in a dataset against the sum of the variance of the squared Procrustes distances for each group within that dataset. In this case, our groups were males and females within either an entire habitat (freshwater or marine) or males and females within sites in each habitat. An index value of zero indicates no differentiation, and increasingly positive values correspond to increased magnitudes of shape differentiation (see Schutz et al. (2009) for details). This index measures differences between groups but more importantly, also adjusts those differences by the collective variances of each group such that increased variance in the sample reduces the measured magnitude of difference between groups and as such, the dimorphism metric varies depending on variance. For the purposes of this study, we consider this adjustment critical as it showcases how variation both within the sexes and across a sample can profoundly affect levels of differentiation.

## 3 RESULTS

### 3.1 Statistical and heuristic assessments of size and shape variation

**FIGURE 2:**
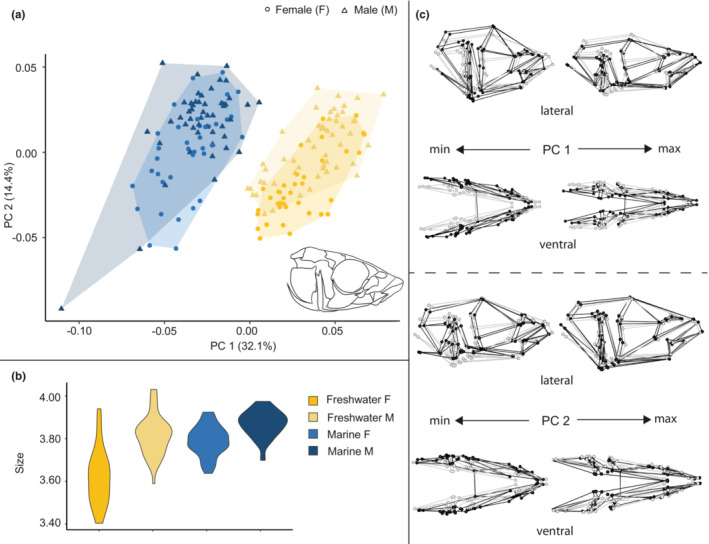
Size and shape analysis of the skull for marine and freshwater habitats. (a) Scatterplot of all specimens on the first two principal components. (b) Violin plot summarizing skull centroid size variation for all specimens. (c) Wireframe deformations describing morphological changes along the first and second principal components with black dots and lines representing the deformation and gray dots and lines representing the mean form.

**FIGURE 3:**
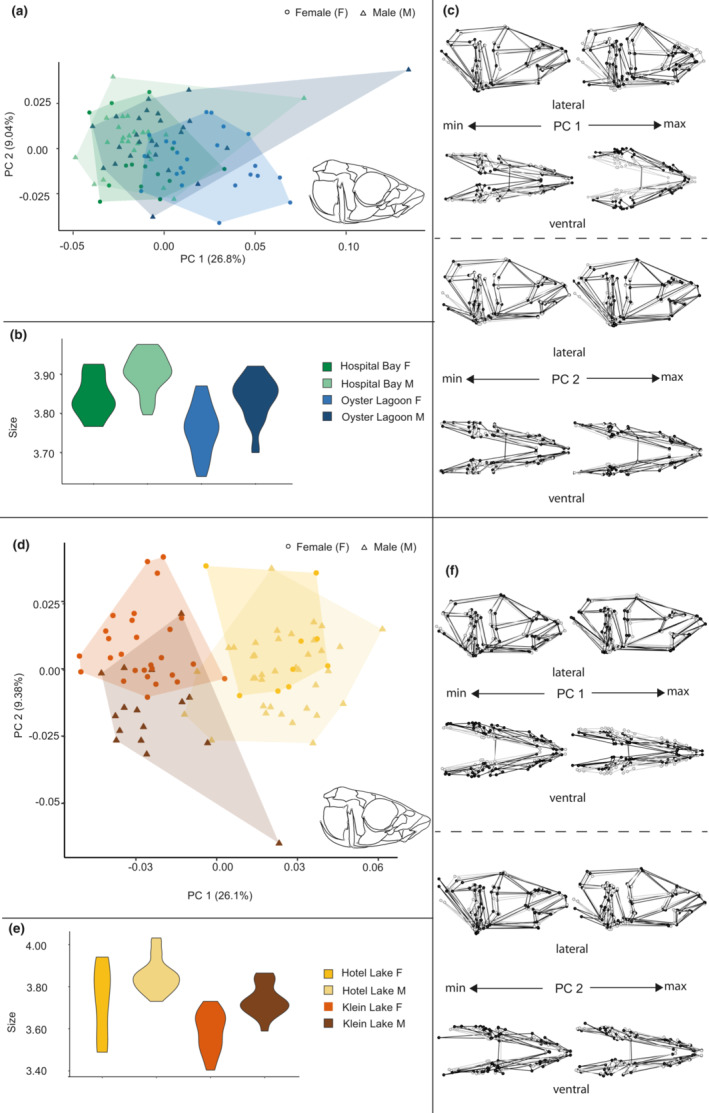
Size and shape analysis of the skull for marine and freshwater sites. (a) Scatterplot of all marine specimens on the first two principal components. (b) Violin plot summarizing skull size variation for marine specimens. (c) Wireframe deformations describing morphological changes in marine specimens along the first and second principal components with black dots and lines representing the deformation and gray dots and lines representing the mean form. (d) Scatterplot of all freshwater specimens on the first two principal components. (e) Violin plot summarizing skull size variation for freshwater specimens. (f) Wireframe deformations describing morphological changes in freshwater specimens along the first and second principal components with black dots and lines representing the deformation and gray dots and lines representing the mean form.

**FIGURE 4:**
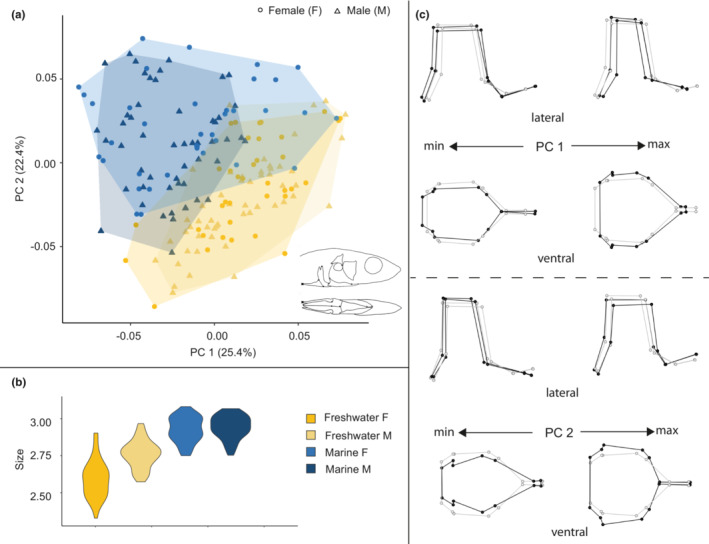
Size and shape analysis of the pectoral girdle for marine and freshwater habitats. (a) Scatterplot of all specimens on the first two principal components. (b) Violin plot summarizing pectoral girdle size variation for all specimens. (c) Wireframe deformations describing morphological changes along the first and second principal components with black dots and lines representing the deformation and gray dots and lines representing the mean form.

**FIGURE 5:**
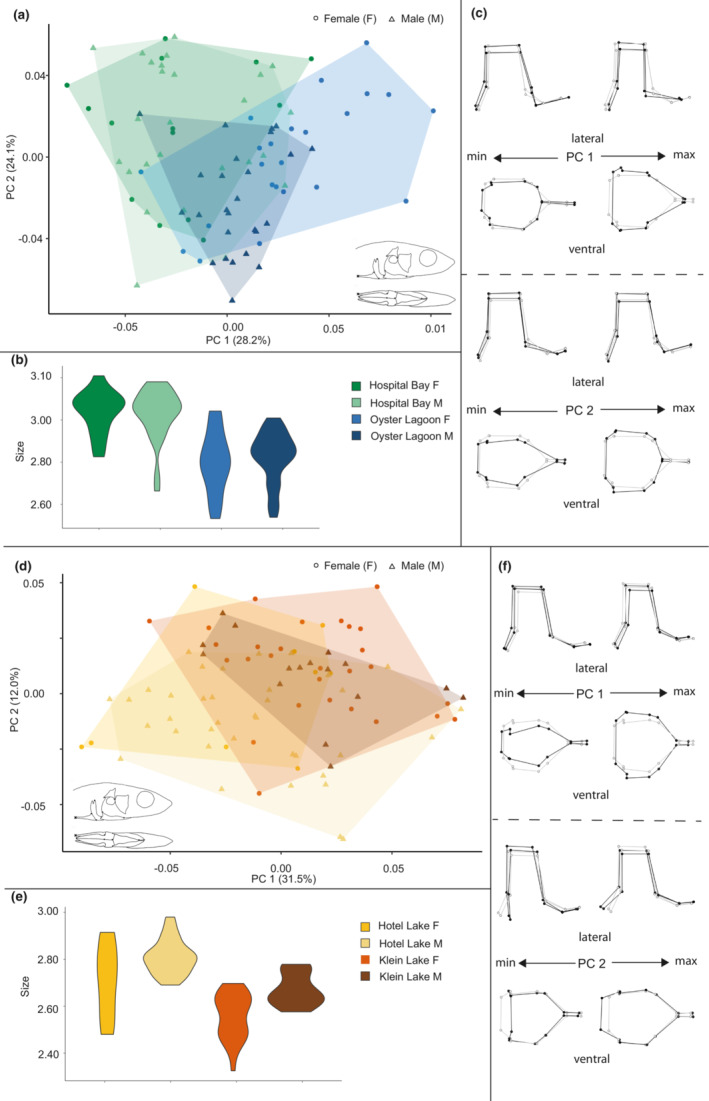
Size and shape analysis of the pectoral girdle for marine and freshwater sites. (a) Scatterplot of all marine specimens on the first two principal components. (b) Violin plot summarizing pectoral girdle size variation for marine specimens. (c) Wireframe deformations describing morphological changes in marine specimens along the first and second principal components with black dots and lines representing the deformation and gray dots and lines representing the mean form. (d) Scatterplot of all freshwater specimens on the first two principal components. (e) Violin plot summarizing pectoral girdle size variation for freshwater specimens. (f) Wireframe deformations describing morphological changes in freshwater specimens along the first and second principal components with black dots and lines representing the deformation and gray dots and lines representing the mean form.

**FIGURE 6:**
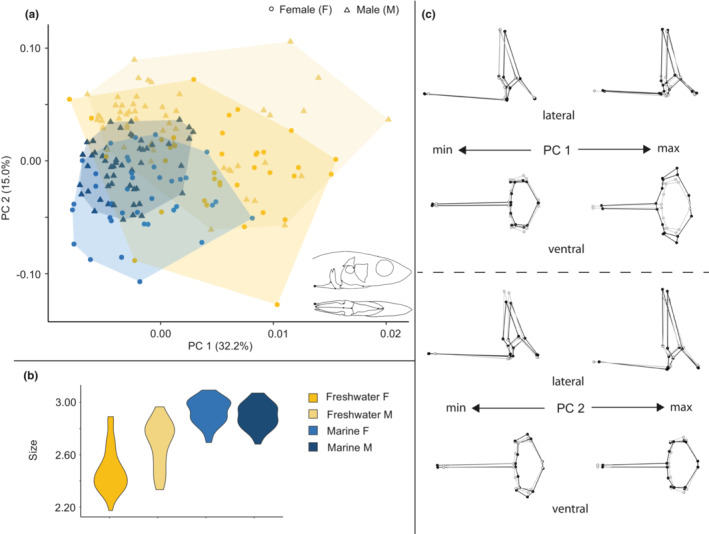
Size and shape analysis of the pelvic girdle for marine and freshwater habitats. (a) Scatterplot of all specimens on the first two principal components. (b) Violin plot summarizing pelvic girdle size variation for all specimens. (c) Wireframe deformations describing morphological changes along the first and second principal components with black dots and lines representing the deformation and gray dots and lines representing the mean form.

**FIGURE 7:**
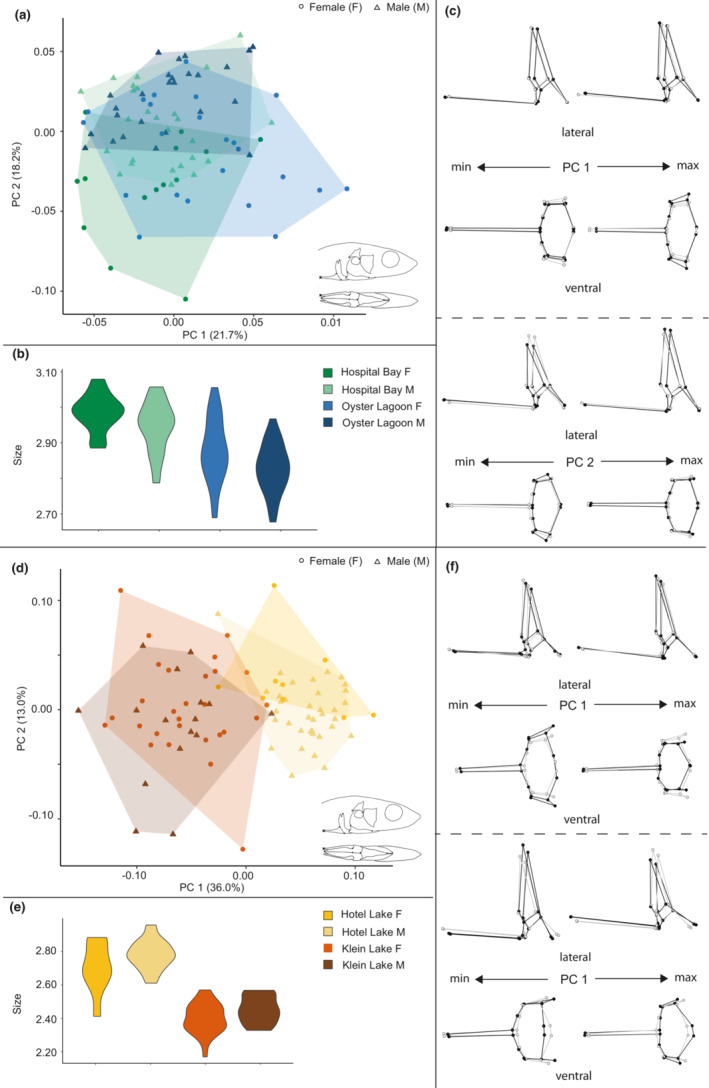
Size and shape analysis of the pelvic girdle for marine and freshwater sites. (a) Scatterplot of all marine specimens on the first two principal components. (b) Violin plot summarizing pelvic girdle size variation for marine specimens. (c) Wireframe deformations describing morphological changes in marine specimens along the first and second principal components with black dots and lines representing the deformation and gray dots and lines representing the mean form. (d) Scatterplot of all freshwater specimens on the first two principal components. (e) Violin plot summarizing pelvic girdle size variation for freshwater specimens. (f) Wireframe deformations describing morphological changes in freshwater specimens along the first and second principal components with black dots and lines representing the deformation and gray dots and lines representing the mean form.

Although size often contributes to shape variation, and many studies attempt to address the effects of size on shape through regression or other standardization approaches (see Klingenberg (2016) for a discussion), we chose not to make any such adjustments here, as the effects of size on shape in this study vary by sex and habitat within each body region. Consequently, size adjustments would require that specific adjustments be made for each subset of the dataset. Instead, we chose to include size as a covariate in our ordination and statistical analyses. Linear models of increasing complexity were evaluated for each dataset to determine the relative contribution of size (as measured by the natural log of landmark configuration centroid size for each element), sex, habitat and site within habitat (analyzed separately) to shape variance. We show the best‐fit models for each dataset in Tables 2, 3, 4 and discuss them below, and provide graphical representations of these results in Figures 2, 3, 4, 5, 6, 7.

**TABLE 2:** Best‐fit ANOVA models for the skull (Figures 2 and 3)

	Element	df	SS	MS	Rsq	*F*	*Z*	*p*
Skull	logSize	1	0.0336	0.0336	0.0597	20.1591	5.6002	.0001
	Sex	1	0.0080	0.0080	0.0142	4.7859	3.1511	.0006
	Habitat	1	0.1731	0.1731	0.3073	103.7449	7.3433	.0001
	Residuals	175	0.2921	0.0017	0.5184			
	Total	178	0.5634					
Marine skull	logSize	1	0.0098	0.0098	0.0581	6.4637	3.0582	.0003
	Sex	1	0.0064	0.0064	0.0380	4.2337	2.5047	.0030
	Site	1	0.0041	0.0041	0.0242	2.6927	1.7522	.0319
	Residuals	85	0.1294	0.0015	0.7636			
	Total	88	0.1695					
Fresh skull	logSize	1	0.0127	0.0127	0.0744	12.2120	4.2471	.0001
	Sex	1	0.0034	0.0034	0.0197	3.2283	2.1341	.0061
	Site	1	0.0373	0.0373	0.2184	35.8629	5.6157	.0001
	Residuals	86	0.0896	0.0010	0.5238			
	Total	89	0.1710					

Abbreviations: Df = degrees of freedom; F = F‐statistic; MS = mean square; *p* = *p*‐value (derived from permutation testing); RSq = R‐squared; SS = sum of squares; Z = effect size.

**TABLE 3:** Best‐fit ANOVA models for the pectoral girdle (Figures 4 and 5)

	Element	df	SS	MS	Rsq	*F*	*Z*	*p*
Pectoral Girdle	logSize	1	0.0140	0.0140	0.0196	4.6554	2.4526	.0019
	Sex	1	0.0123	0.0123	0.0172	4.0897	2.2773	.0048
	Habitat	1	0.0109	0.0109	0.0153	3.6283	2.1279	.0097
	Sex:Habitat	1	0.0077	0.0077	0.0108	2.5740	1.6309	.0389
	Residuals	174	0.5223	0.0030	0.7325			
	Total	178	0.7131					
Marine pectoral girdle	logSize	1	0.0052	0.0052	0.0178	2.0999	1.2599	.0910
	Sex	1	0.0059	0.0059	0.0202	2.3891	1.4578	.0584
	Site	1	0.0186	0.0186	0.0639	7.5501	2.8691	.0003
	Sex:Site	1	0.0073	0.0073	0.0253	2.9882	1.7218	.0288
	Residuals	84	0.2064	0.0025	0.7111			
	Total	88	0.2903					
Fresh pectoral girdle	logSize	1	0.0065	0.0065	0.0192	1.9552	1.3498	.0824
	Sex	1	0.0084	0.0084	0.0248	2.5298	1.7635	.0347
	Site	1	0.0335	0.0335	0.0995	10.1348	3.9407	.0001
	Residuals	86	0.2843	0.0033	0.8444			
	Total	89	0.3367					

Abbreviations: df = degrees of freedom; *F* = *F*‐statistic; MS = mean square; *p* = *p*‐value (derived from permutation testing); RSq = *R*‐squared; SS = sum of squares; *Z* = effect size.

**TABLE 4:** Best‐fit ANOVA models for the pelvic girdle (Figures 6 and 7)

	Element	df	SS	MS	Rsq	*F*	*Z*	*p*
Pelvic girdle	logSize	1	0.0435	0.0435	0.0313	8.9371	3.3979	.0001
	Sex	1	0.0124	0.0124	0.0089	2.5458	1.6698	.0312
	Habitat	1	0.0160	0.0160	0.0115	3.2896	2.0111	.0082
	logSize:Sex	1	0.0106	0.0106	0.0076	2.1732	1.4378	.0601
	logSize:Habitat	1	0.0175	0.0175	0.0125	3.5855	2.1447	.0051
	Sex:Habitat	1	0.0103	0.0103	0.0074	2.1228	1.4108	.0620
	logSize:Sex:Habitat	1	0.0102	0.0102	0.0073	2.0871	1.3930	.0634
	Residuals	171	0.8328	0.0049	0.5985			
	Total	178	1.3914					
Marine pelvic girdle	logSize	1	0.0169	0.0169	0.0425	4.5261	2.6737	.0010
	Sex	1	0.0154	0.0154	0.0389	4.1375	2.5825	.0015
	Site	1	0.0115	0.0115	0.0290	3.0857	2.0813	.0084
	Sex:Site	1	0.0100	0.0100	0.0252	2.6867	1.8901	.0170
	Residuals	84	0.3135	0.0037	0.7889			
	Total	88	0.3974					
Fresh pelvic girdle	logSize	1	0.0181	0.0181	0.0210	3.0075	1.9187	.0118
	Sex	1	0.0272	0.0272	0.0315	4.5177	2.5586	.0005
	Site	1	0.0738	0.0738	0.0855	12.2394	4.0058	.0001
	Residuals	86	0.5183	0.0060	0.6005			
	Total	89	0.8632					

Abbreviations: df = degrees of freedom; *F* = *F*‐statistic; MS = mean square; *p* = *p*‐value (derived from permutation testing); RSq = *R*‐squared; SS = sum of squares; *Z* = effect size.

### 3.2 Skull

The broken stick methodology identified eleven PCs for inclusion in the analysis, including all skulls, and seven PCs for inclusion in each of the marine and freshwater analyses (Table 2). The best fit model for all skulls revealed main effects of size, sex and habitat, and no interactions. We then modeled marine and freshwater skulls separately. Size, sex and site were the main effects in marine skulls, but there were again no significant interactions. The same was true for freshwater skulls, but here, as in other freshwater datasets, site had a much larger effect on shape than did sex.

PC1 in the analysis of all skulls (Figure 2a,c) represented 32.1% of the total variance and described an axis of variation that separated fresh and marine forms into distinct groups. The positive end of the axis was occupied primarily by freshwater individuals and depicted antero‐posteriorly elongated, dorso‐ventrally flattened and medio‐laterally narrowed skulls, whereas the negative end of the axis was occupied primarily by marine individuals and depicted antero‐posteriorly shortened, dorso‐ventrally expanded and more medio‐laterally broadened skulls. PC2 in this analysis represented 14.4% of the total variance and described an axis of variation that somewhat separated males and females, particularly in the marine forms. The subtle shape differences along this axis were primarily in the medio‐lateral breadth, some elements of skull length and dorso‐ventral extent of the skull with females having shorter broader skulls than males and this effect was most pronounced in marine forms.

Plots of skull size trends (Figure 2b) showed that skulls of marine forms were larger than freshwater forms, but that difference was primarily driven by relatively smaller freshwater female skulls.

PC1 in the analysis of marine skulls (Figure 3a,c) represented 26.8% of the total variance and showed minimal differentiation between either the two sites or males and females, although Bargain Bay Lagoon females were the most frequently represented on the positive end of PC1. This axis described variation in which the positive end of the axis was occupied by antero‐posteriorly shorter and medio‐laterally broader skulls, whereas the negative end of the axis was occupied by medio‐laterally narrower and slightly antero‐posteriorly elongated skulls. PC2 in this analysis represented 9.04% of the total variance, provided no differentiation between groups, and described an axis of variation in that is primarily a slight differentiation in medio‐lateral breadth and dorso‐ventral height of the skull.

Size difference trends in marine skulls (Figure 3b) were seen between sites such that Hospital Bay Lagoon individuals tended to be larger than Bargain Bay Lagoon individuals and males tended to be slightly larger than females.

PC1 in the analysis of freshwater skulls (Figure 3d,f) represented 26.1% of the total variance, differentiated between the two freshwater sites and described an axis of variation where antero‐posteriorly longer, dorsoventrally flatter, and medio‐laterally narrower skulls primarily from Hotel Lake individuals occupied the positive end of the axis. Antero‐posteriorly shorter, dorsoventrally more vaulted and medio‐laterally wider skulls, primarily from Klein Lake individuals, occupied the negative end of the axis. PC2 in this analysis represented 9.38% of the total variance and differentiated somewhat between males and females but not uniformly across both sites. The positive and negative ends of the axis showed slight differentiation in the length and dorso‐ventral expansion and compression of the skull, and potentially indicated some minor curvature artifacts in a small number of specimens.

Size difference trends in the skull (Figure 3e) were minimal overall between freshwater sites although males tended to be larger and with more constrained size variation than females.

### 3.3 Pectoral girdle

Six PCs were identified for retention by the broken stick method and retained and included in the analysis including all pectoral girdles, whereas five PCs were retained and included for the marine analysis, and eight were retained and included for the freshwater analysis (Table 3). The best fit model for all pectoral girdles revealed the main effects of size, sex and habitat, and a significant interaction between sex and habitat, confirming differential sexual dimorphism in pectoral girdles. We then modeled marine and freshwater pectoral girdles separately. In marine pectoral girdles, size was a significant main effect but did not interact with other factors, and site was a more important main effect than sex (the latter was not significant). A significant sex‐by‐site interaction indicated that the nature of the effect of sex on shape differed between sites, but we interpret this result with caution given that sex was nearly but not actually significant as a main effect. In freshwater pectoral girdles, size was not a significant main effect, but both sex and site were significant, with site having a greater effect size than sex, and there was no interaction between site and sex.

PC1 in the analysis of all pectoral girdles (Figure 4a,c) represented 25.4%, and PC2 represented 22.4% of the total variance. Individuals on the positive end of PC1 displayed antero‐posterior shortening, dorso‐ventral vaulting and medio‐lateral expansion, whereas individuals on the negative end of this axis displayed antero‐posterior lengthening, slight dorso‐ventral compression and medio‐lateral constriction. Individuals on the positive end of PC2 displayed slight antero‐posterior lengthening, dorso‐ventral compression and medio‐lateral expansion, whereas individuals on the negative end of this axis displayed antero‐posterior shortening, slight dorso‐ventral vaulting and medio‐lateral constriction. Neither axis solely differentiated between marine and freshwater forms, but together, a somewhat distinct freshwater shape space emerged on the positive end of PC 1 and negative end of PC2, whereas marine forms filled the quadrant that was more negative on PC1 and positive on PC2. Differentiation by sex along these two axes was minimal.

Size difference trends in the pectoral girdle (Figure 4b) were seen between marine and freshwater forms such that marine forms were larger than freshwater forms but with minimal sex differences in size. In the freshwater forms, females were smaller than males.

PC1 in the analysis of marine pectoral girdles (Figure 5a,c) represented 28.2% of the total variance and somewhat differentiated between the two marine sites, while providing little to no differentiation between the sexes. The positive end of PC 1 showed anterior–posterior compression and some medio‐lateral expansion of the girdle, while the negative end showed anterior–posterior expansion and mediolateral compression, particularly on the cranial end of the structure. PC2 in this analysis represented 24.1% of the total variance and primarily involved medio‐lateral compression and expansion.

Plots of size difference trends in the pectoral girdle (Figure 5b) between marine sites showed that Hospital Bay Lagoon individuals were larger than those in Bargain Bay Lagoon, but neither shows distinct size differences between the sexes.

PC1 in the analysis of freshwater pectoral girdles (Figure 5d,f) represented 31.5%, and PC2 represented 12% of the total variance. Differentiation between groups occurred primarily along PC2, with Klein Lake clustered more positively and displaying slight anterior–posterior compression and dorso‐ventral vaulting, whereas Hotel Lake clustered more negatively and displayed slight anterior–posterior lengthening and dorso‐ventral shortening.

Plots of size difference trends in the pectoral girdle (Figure 5e) between freshwater sites showed that Hospital Bay Lagoon individuals tended to be larger than those in Bargain Bay Lagoon, but neither site showed distinct sex size differences.

### 3.4 Pelvic girdle

Six PCs were identified for retention via the broken‐stick method and included in the analysis, including all pelvic girdles, while seven PCs were retained and included for the marine analysis and six for the freshwater analysis (Table 4). The best fit model for all pelvic girdles revealed the main effects of size, sex and habitat as well as interactions between size and sex as well as a significant interaction between size and habitat, and a nearly significant interaction between sex and habitat, which we cautiously interpret to indicate the likely presence of differential sexual variation in pelvic girdles. We then modeled marine and freshwater pelvic girdles separately. Size, sex and site were the main effects in marine pelvic girdles, with sex having a slightly larger effect size than site, and there were no interactions between size and other factors. There was a significant interaction between sex and site, indicating differential sexual variation in marine pelvic girdles. In freshwater pelvic girdles, we found main effects of size, sex and site but no significant interactions. As in the pectoral girdle, site had a larger effect than sex on freshwater girdle shape.

PC1 in the analysis of all pelvic girdles (Figure 6a,c) represented 32.2% of the total variance and differentiated between the marine and freshwater forms, primarily due to the more distinctive shape space of marine forms and showed differentiation between freshwater females and males. Along the PC 1 axis of variation, the positive end of the axis was mostly occupied by freshwater individuals and displayed antero‐posterior shortening and medio‐lateral expansion. The negative end of PC1 was occupied primarily by marine individuals and some overlap with freshwater specimens, and displayed antero‐posterior lengthening and medio‐lateral compression. PC2 in this analysis represented 15% of the total variance and described an axis of variation where the positive end of the axis was occupied mostly by males from both groups, with a large number of freshwater males occupying the most extreme positive end of this axis. This axis described slight antero‐posterior shortening, dorso‐ventral expansion and medio‐lateral compression on the positive end and antero‐posterior lengthening, dorso‐ventral compression and medio‐lateral expansion on the negative end.

Plots of size difference trends in the pelvic girdle (Figure 6b) showed that specimens from marine sites were larger than those from freshwater sites. However, only freshwater sites showed size differences between the sexes, where males tended to be larger than females with overlap in size variation between the two.

PC1 in the analysis of marine pelvic girdles (Figure 7a,c) represented 21.7% and PC2 represented 18.2% of the total variance. Neither axis delineated any particular morphospace, with individuals representing both sites and both sexes present in all four quadrants of the plot but clustered primarily negatively on PC1 and positively on PC 2.

Plots of size difference trends in the marine pelvic girdle (Figure 7b) showed that specimens from Hospital Bay Lagoon were larger than those from Bargain Bay Lagoon with some overlap, and minimal size differentiation between the sexes at either site occurred.

PC1 in the analysis of freshwater pelvic girdles (Figure 7d,f) represented 36% of the total variance and separated the Hotel Lake and Klein Lake individuals into distinct groups. The positive end of the PC1 axis displayed slight antero‐posterior expansion and medio‐lateral compression (representing most Hotel Lake individuals). The negative end of the axis displayed antero‐posterior compression and dorso‐ventral and mediolateral expansion. PC2 in this analysis represented 13% of the total variance but did not differentiate either between the two freshwater sites or between the sexes within sites.

Plots of size difference trends in the pelvic girdle (Figure 7e) between freshwater sites showed that specimens from Hotel Lake were larger than those from Klein Lake with minimal size overlap. As with the marine forms, there was minimal size differentiation between the sexes at either site.

### 3.5 Index of sexual dimorphism

Calculating the index of sexual dimorphism for each dataset which adjusts group differences by variance such that greater variance results in a lower differentiation score between groups (Table 5), revealed that overall, the freshwater habitat had greater sexual differentiation than the marine habitat in two of the three regions (skull and pelvic girdle), and the pattern was the opposite for the pectoral girdle, where the marine habitat was more sexually differentiated. This pattern seemed driven by a considerable reduction in sexual differentiation of the pectoral girdle relative to the skull and pelvic girdle in the entire freshwater habitat and within both freshwater sites.

**TABLE 5:** Indices of sexual dimorphism along with mean male‐female Procrustes distances and female and male variance for each dataset

Habitat and sites	Index	Female variance	Male variance	Procrustes distance
*Skull*				
Fresh	0.1584	0.0028	0.0032	0.0309
Klein Lake	0.1641	0.0021	0.0033	0.0298
Hotel Lake	0.1266	0.0028	0.0024	0.0257
Marine	0.1304	0.0030	0.0032	0.0284
Hospital Bay Lagoon	0.0930	0.0026	0.0029	0.0226
Bargain Bay Lagoon	0.1981	0.0027	0.0035	0.0349
*Pectoral girdle*				
Fresh	0.0639	0.0050	0.0044	0.0246
Klein Lake	0.0768	0.0044	0.0041	0.0255
Hotel Lake	0.0846	0.0058	0.0041	0.0290
Marine	0.1354	0.0058	0.0056	0.0407
Hospital Bay Lagoon	0.0902	0.0044	0.0037	0.0271
Bargain Bay Lagoon	0.2878	0.0040	0.0030	0.0450
*Pelvic girdle*				
Fresh	0.1616	0.0135	0.0108	0.0626
Klein Lake	0.1043	0.0121	0.0126	0.0507
Hotel Lake	0.1427	0.0098	0.0053	0.0465
Marine	0.1451	0.0030	0.0032	0.0284
Hospital Bay Lagoon	0.1997	0.0051	0.0051	0.0453
Bargain Bay Lagoon	0.2060	0.0054	0.0058	0.0481

In the skull, the freshwater habitat contained marginally more sexually differentiated individuals than did the marine habitat, but this difference was driven largely by Klein Lake, which was 1.3 times as differentiated as Hotel Lake. When the marine habitat was considered separately, however, marine skulls were also variably differentiated, with Bargain Bay Lagoon having more than twice as much sexual differentiation as Hospital Bay Lagoon. For the pectoral girdle, individuals from marine habitats were more sexually differentiated than those from freshwater habitats, and this difference was again driven by Bargain Bay Lagoon, which was more than three times as differentiated as Hospital Bay Lagoon. For the pelvic girdle, in contrast, the freshwater habitat contained more sexually differentiated individuals, and this difference was driven by Hotel Lake, which was nearly 1.5 times as differentiated as Klein Lake.

## 4 DISCUSSION

In this study, we sought to quantify sexually mediated variation in the three‐dimensional morphology of skull, pectoral and pelvic girdles of threespine sticklebacks, within and between genetic sexes, from replicate sites in both coastal marine and freshwater habitats. We found that the expression of phenotypic variation correlates with our assessment of genetic sex, but is variably impacted by the effects of both habitat (marine vs freshwater) and of individual sites within each habitat. Additionally, relative size exerts variable influence and patterns of phenotypic variation associated with sex vary among body regions. Taken together, these results indicate a complex relationship between genetically inferred sex and environmental factors, supporting our prediction that the interplay between shared genetic background and sexually mediated, ecologically based selective pressures variably structures the phenotypic expression of complex traits. The novelty of this fine‐grained quantification of sexually mediated variation in the context of habitat difference and different anatomical structures lies in the comparisons of how genetic sex structures phenotypic variation under the disparate functional contexts of different morphological structures, sex, and environment at both macro‐ and micro‐levels.

### 4.1 Sexual variation and macro‐level environmental effects

We first examined each body region for the effects of sex and interactions between sex and macro‐level environmental effects, represented by marine or freshwater habitats and including replicate sites within habitats. We found clear differences in skull shape attributable to both habitat and sex (Table 2), with effect sizes indicating that habitat was most important in structuring phenotypic variation but that sex also contributed significantly. Marine forms tend to have shorter, taller and broader skulls than freshwater forms, and there is a distinct separation in morphospace between the two groups. In general, females tend to have somewhat shorter and broader skulls than males, but an overlap exists with male morphology, particularly in the marine environment. The freshwater fish also exhibited the smallest skull centroid size for all females potentially driving the size effect in all body regions given the small size of freshwater females (Figures 2 and 3). Additionally, this freshwater size effect is potentially specifically driven by the relatively smaller Klein Lake fish overall and the specifically small size of Klein Lake female skulls and pectoral girdles (pelvic girdles showed more male–female size overlap). These findings support prior work summarized in the Introduction. We further found that in the skull, freshwater forms had greater sexual differentiation than saltwater forms, both as assessed by our statistical analyses, which demonstrated that morphology varies by habitat and sex, and by the magnitude of the index of sexual dimorphism. This increased level of sexual differentiation in the skulls of freshwater sticklebacks appears driven by Klein Lake and the fact that it tends to have low total variance relative to Hotel Lake, meaning that although the mean male–female Procrustes distances in the two sites are quite similar, the high variance of the Hotel Lake population reduces its index value. These results are congruent with previous work hinting at the complex relationship between sex and phenotypic variation (Caldecutt & Adams, 1998; Pistore et al., 2016, 2019).

In the pectoral girdle, we again found clear differences in phenotype attributable to habitat and sex (Table 3), but here, the two habitats were less clearly differentiated than in the analysis of skull morphology, and effect sizes did not indicate that either factor was more important in structuring variation. At first glance, these findings appear to contradict much prior work showing definitive differences between the sexes (Aguirre et al., 2008; Hoffmann & Borg, 2006; Kitano et al., 2007; Sharpe et al., 2008). Conversely, these results also appear to support other work demonstrating that sexual dimorphism may be substantially reduced in freshwater populations (Albert et al., 2008). Differences in sampling and mode of phenotypic quantification complicate these comparisons, and others have noted the importance of taking a circumspect approach to the interpretation and comparison of morphometric shape differences (Klingenberg, 2013). First, prior observed differences were in linear measurements (sampling either the length or breadth of the fin) of this structure or in shape differences using two two‐dimensional landmarks that sampled the fin as part of a whole‐body landmarking protocol. Our study, in contrast, used seven landmarks per side to sample the structure in three dimensions, and assess it independently from the skull and pelvic girdle landmarks. We did not incorporate these into a whole‐body shape analysis. Consequently, we may be observing patterns not apparent in classical size measurements such as length and area or in the 2D morphometric analyses listed above. Analysis of landmarks focusing solely on one region is also unaffected by variation in landmarks sampling other body regions (when whole body landmarking is used). Since these landmarks sample the structure separately and in greater detail (via a greater number of landmarks in an additional dimension), they ultimately show more subtle sexual differentiation in the overall structure than previously reported.

In the pelvic girdle, we observed a much more complex pattern of variation influenced by both habitat and sex, but even more so by size variation. Here, in contrast to the skull and pectoral girdle morphospaces, the pelvic girdle morphospace was partitioned into a restricted marine region and a much more expanded freshwater region (Figure 6), indicating the presence of greater variation in phenotype among the freshwater specimens. This portion of the analysis therefore provides the first indication that the role of sex, defined by a genetic diagnostic in this paper, is indeed influenced by environmental context and that these interacting effects are not uniform across the organism but rather are potentially subject to a diverse and disparate set of functional constraints.

Prior work shows that pelvic girdle dimensions and presence/absence of the structure in threespine stickleback correlate with lower calcium concentrations and variation in predators (Bell et al., 1993; Bell & Foster, 1994) but appear primarily associated with deletions of the *Pitx1* gene (Chan et al., 2010). However, these reductions, when present, are always found in freshwater forms (Bell et al., 1993). For all of the sites we sampled, we saw no evidence that any group experienced reduction of the pelvic girdle overall, although we did see a trend whereby the pelvic girdle of the freshwater specimens was smaller than that of the saltwater individuals (Figure 6). This size difference is minimal and likely driven by the overall small size of freshwater females across all elements, indicating an overall smaller body size. This pattern also occurs in freshwater males (except in the skull, where they are approximately similar in size to marine males), and as such, freshwater stickleback in this study tended to be smaller overall than marine stickleback.

Our 3D sampling of the pelvic girdle also captured difference trends in the pelvic plate whereby marine forms had shorter pelvic plates relative to freshwater forms even though they have overall smaller pelves, as discussed above. Additionally, our observed shape trends contradict prior work showing that females generally tend to have longer pelvic plates than males (Aguirre et al., 2008; Kitano et al., 2007). However, this contradiction may be due to the considerable amount of age‐related variation seen in this region (Aguirre et al., 2008), a component we did not investigate in this study.

### 4.2 Sexually mediated variation and micro‐level environmental effects

Given our observation that macro‐level environmental effects influenced how sex structured phenotypic variation, we proceeded to examine how local variation, represented here by replicate sites within the same global habitat, potentially contributed to modulating the effects of sex on phenotypic variation. This was accomplished via the examination of different functional contexts as represented by different body regions, where we once again found diverse patterns, speaking to the complexity of the traits and interactions under study.

Across functional regions in threespine sticklebacks, sex tends to exert a greater influence on phenotypic variation within the marine environment, whereas in the freshwater context, micro‐environment (site) is generally more important than sex in structuring phenotypic variation. Our results support previous findings (e.g., Albert et al., 2008; Kitano et al., 2012; Spoljaric & Reimchen, 2008), namely that sexual differentiation patterns vary among different populations within ecologically similar marine and freshwater environments. However, the independent examination of functional traits allowed us to show that variation in sexual differentiation patterns among body regions is mosaic. Further, our results contradict the notion of morphologically conserved marine forms (Bell & Foster, 1994) and add further nuance to our understanding of the patterning of sexual differentiation among ecologically replicate populations, by contributing knowledge about the relative contribution of sex in these different environments.

Earlier work indicated that ecological forces, not sexual selection, primarily drive mean differences between males and females (De Lisle, 2019; De Lisle & Rowe, 2015). Here, we showed that the nature of the relative contribution of ecological and sexually mediated variation fluctuates and is sensitive to context. The well‐studied gene flow effects between proximate marine and freshwater sites consistently showcase a capacity for gene flow to overcome trends toward adaptation to environmental conditions (Ferchaud & Hansen, 2016; Pedersen et al., 2017). Consequently, for marine sites specifically, their even greater reduction of barriers to interchange produces the potential for substantial gene flow among marine sites and likely allows genetic sex to exert a stronger effect on phenotype, whereas in freshwater environments, relatively increased genetic isolation allows environmentally mediated variation to become more prominent. This raises the possibility that the relative contribution of genetic sex to phenotypic variation changes over the course of adaptation to freshwater environments in stickleback, such that genetic sex becomes less and less important as populations become increasingly isolated from one another. A positive feedback loop potentially arises whereby the reduction of the influence of sex accelerates evolution to freshwater environments as populations reach a new phenotypic optimum.

### 4.3 The role of size in structuring sexually mediated variation

The important role of growth in structuring phenotypic variation during organismal development is known (Hallgrímsson et al., 2009), yet its complex effects coupled with incomplete characterization lead to difficulties in generalizing in the face of substantial previous work on the relationship between size and shape in the study of phenotypic variation. Difficulties generalizing occur in part due to the variable effects that size appears to exert on shape in different organismal contexts, making comparisons extremely difficult even within this phylogenetically restricted sampling design that benefits from the capacity for comparison across variable habitats without long divergence times and extreme body size variation. In this study, we made the decision to retain size in our ordinations and statistical analyses, treating it as a covariate to determine if it was affecting shape either on its own or as an interacting effect with other factors.

Our statistical analyses (Tables 2, 3, 4) revealed that element size significantly contributed to overall shape variation in all datasets except the marine and fresh pectoral girdles. Consequently, size contributes to variation in the pectoral dataset potentially due to size differences between fish in the marine and fresh habitats (Figure 5b,e). Additionally, in the macro‐environment datasets, although we did not recover a size by habitat interaction, we found a fluctuating relationship between size and shape depending on body region and habitat.

Within macro‐environments, in marine skulls and pelvic girdles, size had the largest effect on shape. In contrast, for all three elements in the freshwater context, site had a much larger effect on shape than size (or sex). The lack of a size by habitat interaction in the macro‐environment datasets indicated that the variable effect of size on shape revealed in the micro‐environment datasets is subtle and contains a confounding influence attributable to freshwater site and, to a lesser degree, sex.

It is also interesting to note that despite the general overall body size trend in sticklebacks where males tend to be smaller than females (e.g., Kitano et al., 2007), when we partitioned sticklebacks into separate body regions, we found the opposite trend. In all body regions and all sites examined, we found differences in the range of size of females relative to males where female size varied considerably more than male size, yet when mean differences in size were found within sites, males were always larger. Our findings mirror those of other studies showing males having larger skull metrics than females (summary in Kitano et al., 2007), a consistent pattern often associated with male‐attributed breeding and nesting behaviors (Kitano et al., 2007). Our findings for the pectoral and pelvic girdles however, contradict those of other studies. For example, we found that males tend to have larger pectoral girdles than females in freshwater environments, whereas pectoral girdle size hardly differed between the sexes in the marine environment. Keeping in mind that geometric size as computed via centroid size and length are not equivalent, Aguirre et al. (2008) found that females had longer pectoral fins, and as we previously discussed, this difference may be seasonal.

### 4.4 Sexually mediated variation in skeletal traits considered as a continuum

In the present study, we applied a different lens to the assessment of sexually mediated phenotypic variation in complex traits. We chose to avoid a priori assumptions regarding the presence of a strongly bimodal distribution of phenotypes associated with bimodal genetic sex, as is traditionally assumed in such assessments, including via the historical nature of our language and statistical analyses, that inherently use binary codes to classify sex. When examined graphically using this approach, no body region in any habitat showed strong evidence of partitioning of morphospace into regions primarily occupied by one sex or the other. Rather, we showed variable degrees of partitioning of morphospace by habitat or site, but within each environment examined, we found extensive overlap between male and female individuals as determined by the genotyping protocol of Peichel et al. (2004). Although our methods constrained us to a strictly binary description of genotypic sex, here we show that genotypic sex is expressed phenotypically as a continuum that is context dependent.

The results from our assessment using the Dimorphism Index of Schutz et al. (2009) indicated that within macro‐environments, represented by replicate marine and freshwater habitats, the expression of sexually mediated variation itself varies (Table 5). Bargain Bay Lagoon individuals had skulls with twice the level of sexual differentiation as those of Hospital Bay Lagoon individuals, and pectoral girdles that were three times as differentiated. In contrast, the differences in the expression of sexually mediated variation in the freshwater habitat were not nearly as pronounced. The presence of a significant sex by site interaction in the statistical analysis of the marine pectoral girdles (Table 3) further reinforces the presence of strong differential sexual variation in this dataset, as indicated by the index result.

The results of the index analysis support the results of our graphical and statistical analyses, and provide additional context around the presence of sexually mediated variation in the marine environment. We hypothesized above that the presence of relatively greater gene flow in the marine environment allows genetic sex (as we categorized it in this study) to exert a relatively stronger effect on phenotype. Here, we further show that those effects vary within the marine context, and that some marine sites produce a much stronger signal of sexually mediated variation than others due to potential gene flow limiting both genomic and phenotypic diversification (Ferchaud & Hansen, 2016). The effect of gene flow, which is still present in freshwater populations, is less pronounced.

### 4.5 Limitations

Despite long held assumptions regarding the uniformity of marine population ancestry (e.g., Bell & Foster, 1994), recent work implies multiple ancestral marine populations, and that existing marine populations exhibit substantial genetic divergence. These new data show that some freshwater forms share closer relationships with geographically distant marine populations than with the most proximate marine forms (Morris et al., 2018). Patterns of relationships among and within populations from different sites are likely also contributing to observed patterns of phenotypic variation. Further work to produce population‐level phylogenies will allow measured variation to be corrected for patterns of relationship.

We also note that although we reported definitive patterns of phenotypic variation in this contribution, these patterns are subtle, and substantial variance occurs in our dataset at multiple levels including fluctuations in the degree of intrasexual variance in a site, producing fluctuations in mean sex‐differences observed. We also found that each site studied produced a different picture of phenotypic variation. Although this result itself speaks to the complexity of interactions between genetic sex and ecology in structuring phenotypic variation, it indicates that further work making use of larger sample sizes and greater numbers of replicate sites within each habitat type are needed to better characterize the different patterns that are present.

Although beyond the scope of the present contribution, our findings also confirm the importance of future work to consider the effects of potential discrepancies between genotypic and gonadal sex. For this study, we assigned genotypic sex following the well‐accepted protocol of Peichel et al. (2004), but we did not verify gonadal sex via dissection and examination of gonads. Recent work (Toli et al., 2016) suggests a potentially higher error rate for single‐locus assignment of sex, and although it appears that the *Amh* gene on chromosome Y is the master sex‐determination gene in *G. aculeatus* (Peichel et al., 2020), this improved understanding of genetic sex determination in this species does not preclude issues with discordance between genetic sex and gonadal sex or sexual variation in phenotype. For instance, gonadal sex in threespine sticklebacks may be as diverse as that of mammals, at times contradictory to genotypic sex, and exhibit sensitivities to exogenous hormones or their mimics (Lewis et al., 2008). Moreover, gonadally intersex individuals occur in natural populations of *G. aculeatus* (Gercken & Sordyl, 2002), and experimental exposure to relatively low levels of synthetic estrogen (at levels lower than commonly found in the field) produces increased numbers of gonadally intersex fish (Porseryd et al., 2019). Gonadal development in *G. aculeatus* seems to experience deleterious effects in the presence of increased water temperature (Hani et al., 2019). Finally, gonadal morphology is linked to hormone production and whole‐body morphological effects (Petersen et al., 2015). Given all of these considerations, it stands to reason that using genotype as the gold‐standard of sex‐classification may cause us to miss critical sources of variation or to ignore individuals in the zones of overlap.

In conclusion, previous work showed that differential selection regimes between the sexes are associated with different ecological scenarios for each sex within a population (Reimchen et al., 2016) and that strong correlations exist between ecological factors and the magnitude of variation in sexual differentiation (Nosil & Reimchen, 2005; Reimchen et al., 2004) in freshwater sticklebacks. Our results provide additional evidence that the interaction between ecology and genetic sex structures phenotypic variation in a complex and variable fashion across body regions and that this scenario extends beyond freshwater habitats to the marine environment. We demonstrated that the expression of sexually mediated intra‐ and intersexual variation is variable between macro‐environments (freshwater or marine habitat) as well as between micro‐environments (sites within habitat type) in threespine stickleback. Furthermore, we showed that the pattern of sexually mediated variation in the skull and girdles of sticklebacks does not manifest uniformly, but rather as a continuum of variation reflecting the interacting effects of genetic sex and ecology as they act to structure phenotypic variation. We show that considering sexually mediated phenotypic variation through a different lens, explicitly focusing on the extent and variable expression of the range of variation present rather presuming the presence of a biomodal distribution of phenotypes, provides new opportunities to explore the complex relationships between organisms and their environment as they adapt to a rapidly changing world.

## AUTHOR CONTRIBUTIONS

**Heidi Schutz:** Conceptualization (lead); data curation (lead); formal analysis (lead); funding acquisition (lead); investigation (equal); methodology (lead); project administration (lead); resources (equal); software (equal); supervision (lead); validation (lead); visualization (equal); writing – original draft (lead); writing – review and editing (lead). **Rebecca J. Anderson:** Conceptualization (supporting); data curation (supporting); formal analysis (supporting); funding acquisition (supporting); investigation (equal); methodology (supporting); project administration (supporting); software (supporting); writing – review and editing (supporting). **Ethan G. Warwick:** Conceptualization (supporting); data curation (supporting); formal analysis (supporting); investigation (equal); methodology (supporting). **Tegan N. Barry:** Data curation (equal); investigation (equal); methodology (supporting); writing – review and editing (supporting). **Heather A. Jamniczky:** Conceptualization (lead); data curation (lead); formal analysis (lead); funding acquisition (lead); investigation (equal); methodology (lead); project administration (lead); resources (lead); software (lead); supervision (lead); validation (lead); visualization (lead); writing – original draft (equal); writing – review and editing (equal).

## CONFLICT OF INTEREST

None of the authors have any conflicts of interest to report.

### DATA AVAILAIBILITY STATEMENT

The raw landmark data from scans and R code used in all analyses are archived in Dryad and accessible here: https://doi.org/10.5061/dryad.xd2547dkw.

### OPEN RESEARCH BADGES

This article has earned an Open Data badge for making publicly available the digitally‐shareable data necessary to reproduce the reported results. The data is available at https://doi.org/10.5061/dryad.xd2547dkw.

## References

[ece39367-bib-0001] Aguirre, W. E. , Ellis, K. E. , Kusenda, M. , & Bell, M. A. (2008). Phenotypic variation and sexual dimorphism in anadromous threespine stickleback: Implications for postglacial adaptive radiation. Biological Journal of the Linnean Society, 95(3), 465–478. 10.1111/j.1095-8312.2008.01075.x

[ece39367-bib-0002] Albert, A. Y. K. , Sawaya, S. , Vines, T. H. , Knecht, A. K. , Miller, C. T. , Summers, B. R. , Balabhadra, S. , Kingsley, D. M. , & Schluter, D. (2008). The genetics of adaptive shape shift in stickleback: Pleiotropy and effect size. Evolution, 62(1), 76–85. 10.1111/j.1558-5646.2007.00259.x 18005154

[ece39367-bib-0003] Baker, J. A. , Wund, M. A. , Heins, D. C. , King, R. W. , Reyes, M. L. , & Foster, S. A. (2015). Life‐history plasticity in female threespine stickleback. Heredity, 115(4), 322–334. 10.1038/hdy.2015.65 26286665 PMC4815461

[ece39367-bib-0004] Barry, T. N. (2019). Ecology and genetics of phenotypic integration and the role for adaptation in threespine stickleback. PhD Thesis, University of Calgary.

[ece39367-bib-0005] Bell, M. A. , & Foster, S. A. (1994). The evolutionary biology of the threespine stickleback. Oxford University Press.

[ece39367-bib-0006] Bell, M. A. , Ortí, G. , Walker, J. A. , & Koenings, J. P. (1993). Evolution of pelvic reduction in threespine stickleback fish: A test of competing hypotheses. Evolution, 47(3), 906–914. 10.1111/j.1558-5646.1993.tb01243.x 28567888

[ece39367-bib-0007] Berns, C. M. (2013). The evolution of sexual dimorphism: Understanding mechanisms of sexual shape differences. In Moriyama H. (Ed.), Sexual dimorphism (pp. 1–16). IntechOpen.

[ece39367-bib-0008] Bolnick, D. I. , & Doebeli, M. (2003). Sexual dimorphism and adaptive speciation: Two sides of the same ecological coin. Evolution, 57(11), 2433–2449. 10.1111/j.0014-3820.2003.tb01489.x 14686521

[ece39367-bib-0009] Butler, M. A. , & Losos, J. B. (2002). Multivariate sexual dimorphism, sexual selection, and adaptation in greater Antillean Anolis lizards. Ecological Monographs, 72(4), 541–559.

[ece39367-bib-0010] Butler, M. A. , Sawyer, S. A. , & Losos, J. B. (2007). Sexual dimorphism and adaptive radiation in Anolis lizards. Nature, 447(7141), 202–205. 17495925 10.1038/nature05774

[ece39367-bib-0011] Caldecutt, W. J. , & Adams, D. C. (1998). Morphometrics of trophic osteology in the threespine stickleback, Gasterosteus aculeatus. Copeia, 1998(4), 827–838.

[ece39367-bib-0012] Chan, Y. F. , Marks, M. E. , Jones, F. C. , Villarreal, G., Jr. , Shapiro, M. D. , Brady, S. D. , Southwick, A. M. , Absher, D. M. , Grimwood, J. , Schmutz, J. , Myers, R. M. , Petrov, D. , Jónsson, B. , Schluter, D. , Bell, M. A. , & Kingsley, D. M. (2010). Adaptive evolution of pelvic reduction in sticklebacks by recurrent deletion of a Pitx1 enhancer. Science, 327(5963), 302–305. 10.1126/science.1182213 20007865 PMC3109066

[ece39367-bib-0013] Collyer, M. L. , & Adams, D. C. (2018). RRPP: An r package for fitting linear models to high‐dimensional data using residual randomization. Methods in Ecology and Evolution, 9(7), 1772–1779.

[ece39367-bib-0014] Collyer, M. L. , Sekora, D. J. , & Adams, D. C. (2015). A method for analysis of phenotypic change for phenotypes described by high‐dimensional data. Heredity, 115(4), 357–365. 10.1038/hdy.2014.75 25204302 PMC4815463

[ece39367-bib-0015] Cooper, I. A. , Gilman, R. T. , & Boughman, J. W. (2011). Sexual dimorphism and speciation on two ecological coins: Patterns from nature and theoretical predictions. Evolution, 65(9), 2553–2571. 10.1111/j.1558-5646.2011.01332.x 21884056

[ece39367-bib-0016] Dalziel, A. C. , Vines, T. H. , & Schulte, P. M. (2012). Reductions in prolonged swimming capacity following freshwater colonization in multiple threespine stickleback populations. Evolution, 66(4), 1226–1239. 10.1111/j.1558-5646.2011.01498.x 22486700

[ece39367-bib-0017] Darwin, C. (1859). On the origin of species: A facsimile of the (1st ed.). Harvard University Press.

[ece39367-bib-0018] De Lisle, S. P. (2019). Understanding the evolution of ecological sex differences: Integrating character displacement and the Darwin‐Bateman paradigm. Evolution Letters, 3(5), 434–447. 10.1002/evl3.134

[ece39367-bib-0019] De Lisle, S. P. , & Rowe, L. (2015). Ecological character displacement between the sexes. The American Naturalist, 186(6), 693–707. 10.1086/683775 26655977

[ece39367-bib-0020] Dryden, I. L. , & Mardia, K. V. (1998). Statistical shape analysis: Wiley series in probability and statistics. John Wiley & Sons, Ltd.

[ece39367-bib-0021] Fairbairn, D. J. , & Preziosi, R. F. (1994). Sexual selection and the evolution of allometry for sexual size dimorphism in the water strider, *Aquarius remigis* . The American Naturalist, 144(1), 101–118.

[ece39367-bib-0022] Ferchaud, A.‐L. , & Hansen, M. M. (2016). The impact of selection, gene flow and demographic history on heterogeneous genomic divergence: Three‐spine sticklebacks in divergent environments. Molecular Ecology, 25(1), 238–259. 10.1111/mec.13399 26412233

[ece39367-bib-0023] Gercken, J. , & Sordyl, H. (2002). Intersex in feral marine and freshwater fish from northeastern Germany. Marine Environmental Research, 54(3), 651–655. 10.1016/S0141-1136(02)00156-3 12408630

[ece39367-bib-0024] Hallgrímsson, B. , Jamniczky, H. , Young, N. M. , Rolian, C. , Parsons, T. E. , Boughner, J. C. , & Marcucio, R. S. (2009). Deciphering the palimpsest: Studying the relationship between morphological integration and phenotypic covariation. Evolutionary Biology, 36(4), 355–376. 10.1007/s11692-009-9076-5 23293400 PMC3537827

[ece39367-bib-0025] Hani, Y. M. I. , Turies, C. , Palluel, O. , Delahaut, L. , Bado‐Nilles, A. , Geffard, A. , Dedourge‐Geffard, O. , & Porcher, J.‐M. (2019). Effects of a chronic exposure to different water temperatures and/or to an environmental cadmium concentration on the reproduction of the threespine stickleback (*Gasterosteus aculeatus*). Ecotoxicology and Environmental Safety, 174, 48–57. 10.1016/j.ecoenv.2019.02.032 30818260

[ece39367-bib-0026] Hendry, A. P. , Peichel, C. L. , Matthews, B. , Boughman, J. W. , & Nosil, P. (2013). Stickleback research: The now and the next. Evolutionary Ecology Research, 15(2), 111–141.

[ece39367-bib-0027] Hoffmann, E. , & Borg, B. (2006). Sex differences in pectoral muscles but not in pectoral fins in the three‐spined stickleback *Gasterosteus aculeatus* . Journal of Fish Biology, 68(5), 1451–1459. 10.1111/j.0022-1112.2006.001030.x

[ece39367-bib-0028] Jackson, D. A. (1993). Stopping rules in principal components analysis: A comparison of heuristical and statistical approaches. Ecology, 74(8), 2204–2214.

[ece39367-bib-0029] Jamniczky, H. A. , Barry, T. N. , & Rogers, S. M. (2015). Eco‐evo‐devo in the study of adaptive divergence: Examples from Threespine stickleback (*Gasterosteus aculeatus*). Integrative and Comparative Biology, 55(1), 166–178. 10.1093/icb/icv018 25908668

[ece39367-bib-0030] Kimmel, C. B. , Cresko, W. A. , Phillips, P. C. , Ullmann, B. , Currey, M. , von Hippel, F. , Kristjánsson, B. K. , Gelmond, O. , & McGuigan, K. (2012). Independent axes of genetic variation and parallel evolutionary divergence of opercle bone shape in threespine stickleback. Evolution, 66(2), 419–434. 10.1111/j.1558-5646.2011.01441.x 22276538 PMC4039416

[ece39367-bib-0031] Kitano, J. , Mori, S. , & Peichel, C. L. (2007). Sexual dimorphism in the external morphology of the Threespine stickleback (*Gasterosteus aculeatus*). Copeia, 2007(2), 336–349. 10.1643/0045-8511(2007)7[336:SDITEM]2.0.CO;2

[ece39367-bib-0032] Kitano, J. , Mori, S. , & Peichel, C. L. (2012). Reduction of sexual dimorphism in stream‐resident forms of three‐spined stickleback *Gasterosteus aculeatus* . Journal of Fish Biology, 80(1), 131–146. 10.1111/j.1095-8649.2011.03161.x 22220894 PMC3847934

[ece39367-bib-0033] Klepaker, T. O. , Østbye, K. , & Bell, M. A. (2013). Regressive evolution of the pelvic complex in stickleback fishes: A study of convergent evolution. Evoltionary Ecology Research, 15(4), 413–435.

[ece39367-bib-0034] Klingenberg, C. P. (2013). Visualizations in geometric morphometrics: How to read and how to make graphs showing shape changes. Hystrix, 24(1), 15.

[ece39367-bib-0035] Klingenberg, C. P. (2016). Size, shape, and form: Concepts of allometry in geometric morphometrics. Development Genes and Evolution, 226(3), 113–137. 10.1007/s00427-016-0539-2 27038023 PMC4896994

[ece39367-bib-0036] Künzler, R. , & Bakker, T. C. M. (2000). Pectoral fins and paternal quality in sticklebacks. Proceedings of the Royal Society of London. Series B: Biological Sciences, 267(1447), 999–1004. 10.1098/rspb.2000.1102 PMC1690627 10874749

[ece39367-bib-0037] Lande, R. (1980). Sexual dimorphism, sexual selection, and adaptation in polygenic characters. Evolution, 34(2), 292–305. 10.2307/2407393 28563426

[ece39367-bib-0038] Legendre, P. , & Legendre, L. (2012). Numerical Ecology (Vol. 24). Elsevier.

[ece39367-bib-0039] Leinonen, T. , Cano, J. M. , MÄKinen, H. , & MerilÄ, J. (2006). Contrasting patterns of body shape and neutral genetic divergence in marine and lake populations of threespine sticklebacks. Journal of Evolutionary Biology, 19(6), 1803–1812. 10.1111/j.1420-9101.2006.01182.x 17040377

[ece39367-bib-0040] Leinonen, T. , Cano, J. M. , & Merila, J. (2011). Genetic basis of sexual dimorphism in the threespine stickleback *Gasterosteus aculeatus* . Heredity, 106(2), 218–227. 20700139 10.1038/hdy.2010.104 PMC3183875

[ece39367-bib-0041] Lewis, Z. R. , McClellan, M. C. , Postlethwait, J. H. , Cresko, W. A. , & Kaplan, R. H. (2008). Female‐specific increase in primordial germ cells marks sex differentiation in threespine stickleback (*Gasterosteus aculeatus*). Journal of Morphology, 269(8), 909–921. 10.1002/jmor.10608 18157863 PMC7220811

[ece39367-bib-0042] MacLeod, N. , & Kolska Horwitz, L. (2020). Machine‐learning strategies for testing patterns of morphological variation in small samples: Sexual dimorphism in gray wolf (*Canis lupus*) crania. BMC Biology, 18(1), 113. 10.1186/s12915-020-00832-1 32883273 PMC7470621

[ece39367-bib-0043] McGee, M. D. , & Wainwright, P. C. (2013). Sexual dimorphism in the feeding mechanism of threespine stickleback. Journal of Experimental Biology, 216(5), 835–840. 10.1242/jeb.074948 23408802

[ece39367-bib-0044] McKinnon, J. S. , & Rundle, H. D. (2002). Speciation in nature: The threespine stickleback model systems. Trends in Ecology & Evolution, 17(10), 480–488.

[ece39367-bib-0045] Morris, M. R. J. , Bowles, E. , Allen, B. E. , Jamniczky, H. A. , & Rogers, S. M. (2018). Contemporary ancestor? Adaptive divergence from standing genetic variation in Pacific marine threespine stickleback. BMC Evolutionary Biology, 18(1), 113. 10.1186/s12862-018-1228-8 30021523 PMC6052716

[ece39367-bib-0046] Nosil, P. , & Reimchen, T. E. (2005). Ecological opportunity and levels of morphological variance within freshwater stickleback populations. Biological Journal of the Linnean Society, 86(3), 297–308. 10.1111/j.1095-8312.2005.00517.x

[ece39367-bib-0047] Oksanen, J. , Blanchet, F. G. , Friendly, M. , Kindt, R. , Legendre, P. , McGlinn, D. , Minchin, P. R. , O'Hara, B. , Simpson, G.L. , Solymos, P. , Stevens, M.H.H. , Szoecs, E. , Wagner, H. (2019). Vegan: Community ecology package. Retrieved from https://CRAN.R‐project.org/package=vegan

[ece39367-bib-0048] Pedersen, S. H. , Ferchaud, A.‐L. , Bertelsen, M. S. , Bekkevold, D. , & Hansen, M. M. (2017). Low genetic and phenotypic divergence in a contact zone between freshwater and marine sticklebacks: Gene flow constrains adaptation. BMC Evolutionary Biology, 17(1), 130. 10.1186/s12862-017-0982-3 28587593 PMC5461706

[ece39367-bib-0049] Peichel, C. L. , McCann, S. R. , Ross, J. A. , Naftaly, A. F. S. , Urton, J. R. , Cech, J. N. , Grimwood, J. , Schmutz, J. , Myers, R. M. , Kingsley, D. M. , & White, M. A. (2020). Assembly of the threespine stickleback Y chromosome reveals convergent signatures of sex chromosome evolution. Genome Biology, 21(1), 177. 10.1186/s13059-020-02097-x 32684159 PMC7368989

[ece39367-bib-0050] Peichel, C. L. , Ross, J. A. , Matson, C. K. , Dickson, M. , Grimwood, J. , Schmutz, J. , Myers, R. M. , Mori, S. , Schluter, S. , & Kingsley, D. M. (2004). The master sex‐determination locus in threespine sticklebacks is on a nascent Y chromosome. Current Biology, 14, 1416–1424. 10.1016/j.cub.2004.08.030 15324658

[ece39367-bib-0051] Petersen, A. M. , Dillon, D. , Bernhardt, R. R. , Torunsky, R. , Postlethwait, J. H. , von Hippel, F. A. , Loren Buck, C. , & Cresko, W. A. (2015). Perchlorate disrupts embryonic androgen synthesis and reproductive development in threespine stickleback without changing whole‐body levels of thyroid hormone. General and Comparative Endocrinology, 210, 130–144. 10.1016/j.ygcen.2014.10.015 25448260 PMC4280913

[ece39367-bib-0052] Pistore, A. , Barry, T. N. , Bowles, E. , Sharma, R. , Vanderzwan, S. , Rogers, S. M. , & Jamniczky, H. A. (2016). Characterizing phenotypic divergence using three‐dimensional geometric morphometrics in four populations of threespine stickleback (*Gasterosteus aculeatus*; Pisces: Gasterosteidae) in Katmai National Park and preserve, Alaska. Canadian Journal of Zoology, 94(7), 463–472.

[ece39367-bib-0053] Pistore, A. E. , Barry, T. N. , Vanderzwan, S. L. , Schutz, H. , Rogers, S. M. , & Jamniczky, H. A. (2019). Ontogeny and allometry of habitat‐specific phenotypic variation in complex phenotypes in the Threespine stickleback (*Gasterosteus aculeatus* L.). Evolutionary Ecology Research, 20, 27–50.

[ece39367-bib-0054] Porseryd, T. , Larsson, J. , Kellner, M. , Bollner, T. , Dinnétz, P. , & Porsch Hällström, I. (2019). Altered non‐reproductive behavior and feminization caused by developmental exposure to 17α‐ethinylestradiol persist to adulthood in three‐spined stickleback (*Gasterosteus aculeatus*). Aquatic Toxicology, 207, 142–152. 10.1016/j.aquatox.2018.11.024 30572174

[ece39367-bib-0055] Punzalan, D. , & Hosken, D. J. (2010). Sexual dimorphism: Why the sexes are (and are not) different. Current Biology, 20(22), R972–R973. 10.1016/j.cub.2010.09.067 21093787

[ece39367-bib-0056] RStudio Team (2020). RStudio: Integrated Development for R. RStudio, PBC. http://www.rstudio.com/

[ece39367-bib-0057] R Team . (2020). R: A language and environment for statistical computing. R Foundation for Statistical Computing. Retrieved from. http://www.R‐project.org/

[ece39367-bib-0058] Reimchen, T. E. , Nosil, P. , & Wainwright, P. (2004). Variable predation regimes predict the evoluiton of sexual dimoprhism in a population of threespine stickleback. Evolution, 58(6), 1274–1281. 10.1554/03-558 15266976

[ece39367-bib-0059] Reimchen, T. , Steeves, D. , & Bergstrom, C. (2016). Sex matters for defence and trophic traits of threespine stickleback. Evolutionary Ecology Research, 17(4), 459–485.

[ece39367-bib-0060] Ronco, F. , Roesti, M. , & Salzburger, W. (2019). A functional trade‐off between trophic adaptation and parental care predicts sexual dimorphism in cichlid fish. Proceedings of the Royal Society B, 286(1909), 20191050. 31431167 10.1098/rspb.2019.1050 PMC6732390

[ece39367-bib-0062] Schutz, H. , Polly, P. D. , Krieger, J. D. , & Guralnick, R. P. (2009). Differential sexual dimorphism: Size and shape in the cranium and pelvis of gray foxes (*Urocyon*). Biological Journal of the Linnean Society, 96(2), 339–353.

[ece39367-bib-0063] Sharpe, D. M. , Räsänen, K. , Berner, D. , & Hendry, A. P. (2008). Genetic and environmental contributions to the morphology of lake and stream stickleback: Implications for gene flow and reproductive isolation. Evolutionary Ecology Research, 10(6), 849–866.

[ece39367-bib-0064] Slatkin, M. (1984). Ecological causes of sexual dimorphism. Evolution, 38, 622–630. 28555984 10.1111/j.1558-5646.1984.tb00327.x

[ece39367-bib-0065] Spoljaric, M. A. , & Reimchen, T. E. (2008). Habitat‐dependent reduction of sexual dimorphism in geometric body shape of Haida Gwaii threespine stickleback. Biological Journal of the Linnean Society, 95(3), 505–516. 10.1111/j.1095-8312.2008.01068.x

[ece39367-bib-0066] Svanbäck, R. , & Schluter, D. (2012). Niche specialization influences adaptive phenotypic plasticity in the threespine stickleback. The American Naturalist, 180(1), 50–59. 10.1086/666000 22673650

[ece39367-bib-0067] Temeles, E. J. , Miller, J. S. , & Rifkin, J. L. (2010). Evolution of sexual dimorphism in bill size and shape of hermit hummingbirds (Phaethornithinae): A role for ecological causation. Philosophical Transactions of the Royal Society B: Biological Sciences, 365(1543), 1053–1063. 10.1098/rstb.2009.0284 PMC2830232 20194168

[ece39367-bib-0068] Toli, E. A. , Calboli, F. C. F. , Shikano, T. , & Merilä, J. (2016). A universal and reliable assay for molecular sex identification of three‐spined sticklebacks (*Gasterosteus aculeatus*). Molecular Ecology Resources, 16(6), 1389–1400. 10.1111/1755-0998.12543 27238091

[ece39367-bib-0069] Webster, M. M. , Atton, N. , Hart, P. J. B. , & Ward, A. J. W. (2011). Habitat‐specific morphological variation among threespine sticklebacks (*Gasterosteus aculeatus*) within a drainage basin. PLoS One, 6(6), e21060. 10.1371/journal.pone.0021060 21698269 PMC3115991

[ece39367-bib-0070] Wickham, H. (2011). The split‐apply‐combine strategy for data analysis. Journal of Statistical Software, 40(1), 1–29.

[ece39367-bib-0071] Wickham, H. (2016). ggplot2: Elegant graphics for data analysis. Springer‐Verlag.

[ece39367-bib-0072] Willacker, J. J. , Von Hippel, F. A. , Wilton, P. R. , & Walton, K. M. (2010). Classification of threespine stickleback along the benthic–limnetic axis. Biological Journal of the Linnean Society, 101(3), 595–608. 21221422 10.1111/j.1095-8312.2010.01531.x PMC3017379

